# Unified continental approach to mpox preparedness and response: A model for Africa’s future outbreaks

**DOI:** 10.4102/jphia.v16i1.877

**Published:** 2025-03-31

**Authors:** Ngashi Ngongo, Nicaise Ndembi, Mosoka Fallah, Wessam Mankoula, Merawi A. Tegegne, Moréniké Oluwátóyìn Foláyan, Jean Kaseya

**Affiliations:** 1 Africa Centres for Disease Control and Prevention (Africa CDC), Addis Ababa, Ethiopia; 2 Department of Child Dental Health, Obafemi Awolowo University, Ile-Ife, Nigeria

Less than 2 years after the World Health Organization (WHO) declared the end of the sixth Public Health Emergency of International Concern (PHEIC), mpox has resurfaced globally with a novel clade 1b virus, primarily spread through human-to-human physical and sexual contact, and disproportionately affecting children and key populations.^1,2^ Originating in Democratic Republic of Congo (DRC), the outbreak had spread to Burundi, Kenya, Rwanda, Uganda, and more recently, to India.^3,4^ The rise in cases, associated mortality and spread to non-endemic countries, prompted the Africa Centres for Disease Control and Prevention (Africa CDC) and WHO to declare the outbreak a Public Health Emergency of Continental Security (PHECS) and PHEIC, respectively.^5,6,7^

Despite these declarations, cases continue to rise. By epidemiological week 39 of 2024, 35 525 cases have been reported across 16 African Union Member States, with 6754 confirmed and 996 deaths. Between week 33 and week 39, 14 countries have reported 11 933 new cases, including 1810 confirmed cases, with DRC, Burundi and Nigeria being the most affected. The Democratic Republic of Congo and Burundi account for 94% of the cases.^8^

While the PHECS and PHEIC are expected to enhance the mobilisation of resources to support the response and enhance international collaboration for outbreak containment, the containment of the outbreak will depend on swift implementation of response strategies and effective public health interventions, particularly in high-risk areas. The goal of the Africa CDC and its partners is to respond to the mpox outbreak in a strategic, cost-effective and impactful way by building on the recommended five strategic shifts of the Lusaka Agenda for transforming the global health ecosystem and optimise health impacts.^9^ The agenda emphasised that unified and coordinated actions are crucial to accelerate health improvements across the continent.

The continental mpox Incident Management Support Team (IMST) was established to coordinate the mpox outbreak response actions in Africa. It adopted the unified ‘4 ONEs’ approach – One Team, One Plan, One Budget and One Monitoring and Evaluation (M&E) Framework – as the framework of the mpox preparedness and response strategy. This approach aims to streamline coordination, optimise resource utilisation and ensure the alignment of efforts across countries and sectors to effectively control the outbreak.

## One team

The IMST was established under the co-leadership of Africa CDC and WHO to mobilise partners for a coordinated continental response. The collaboration currently has 26 partners, each contributing to different areas of the response based on their expertise. For example, disease surveillance is supported by Africa CDC, International Organization for Migration (IOM) manages port of entry surveillance and WHO manages health facility-based surveillance and the integrated disease surveillance and response. Clinical case management is led by Médecins Sans Frontières (MSF) and WHO, United Nations International Children’s Emergency Fund (UNICEF) and World Food Programme (WFP) handle nutritional support, and mental health services are managed by Africa CDC and UNICEF. The Africa CDC-WHO co-led IMST is the central coordinating body that provides technical support to affected countries, and manages the distribution of critical medical countermeasures.

This coordinated approach ensures countries receive support from the most experienced organisations. Regular communication, including weekly teleconferences with national public health institutes, donors, partners and the media, keeps stakeholders updated on the epidemiological situation and response efforts. In addition, the continental IMST constituted a technical review committee (TRC) of global experts drawn from partner institutions and organisations, and from academic institutions to review country plans and advise on the allocation of vaccines, diagnostics and other medical resources.

## One plan

A unified Continental Mpox Preparedness and Response Plan (CMPRP) has been established to guide efforts from September 2024 to February 2025. This 6-month plan includes both response activities for the 14 affected countries in Africa and preparedness measures for the 15 most at-risk nations.

It focusses on 10 critical response pillars: coordination and collaboration, risk communication and community engagement (RCCE), disease surveillance, laboratory testing and sequencing, infection prevention and control (IPC), vaccination, case management, continuity of services, research and innovation, and logistics.

To optimise resources and in alignment with the Lusaka agenda, partners have committed to supporting specific pillars of the CMPRP thereby creating a division of labour among key partners, ensuring greater synergy and efficiency and effective use of allocated resources. Identifying and addressing gaps for under-resourced areas of the response is a lot easier this way.

## One budget

The unified 6-month mpox response plan includes a budget of $599 million: $330 million to support 29 affected and at-risk countries, and $269 million for technical support from key partners to strengthen national preparedness and response capacities. Vaccine procurement is expected to rely on international donations. Budget allocations are as follows: $77 million (24.4%) for vaccination, $55 million (17.6%) for case management, $41 million (13.0%) for IPC, $39 million (12.4%) for surveillance, $39 million (12.2%) for RCCE, $36 million (11.5%) for research and innovations, $16 million (5.0%) for laboratory testing and sequencing, and $12 million (3.9%) for coordination.

The plan anticipates a peak caseload of 4000 cases per week in the early months, gradually declining as the outbreak is controlled. The target is to vaccinate 92 000 cases and 10 million people who are members of priority population like healthcare workers, case contacts and vulnerable populations.^10^

The international community’s support has been substantial: $314 million was pledged by partners at the Africa CDC’s meeting, and the United States (US) committed $500 million along with 1 million MVA-BN (Modified Vaccinia Ankara-Bavarian Nordic) vaccine doses. In addition, African countries like the DRC, Burundi, Ivory Coast and Rwanda have contributed financially.

## One monitoring and evaluation framework

A results-based management approach was adopted to establish key performance indicators (KPIs), enabling clear tracking of each strategic objective in the unified CMPRP, meeting the needs of key stakeholders and donors. The framework consists of 20 KPIs, each with defined targets, verification methods and reporting frequency. These KPIs align with Africa CDC’s newly published mpox surveillance reporting protocol – developed with input from affected countries and partners – and WHO’s interim guidance on surveillance, case investigation and contact tracing.^11,12^

To ensure transparency and accountability, a dashboard tracking contributions (human resources, funding, vaccines) from all sources was developed. The tracking facilitates openness and fairness in resource distribution with priority given to those most in need. In addition, a joint weekly situation report is produced to provide updates on the epidemiological status and progress of the response for stakeholders.

## Paving the way for effective outbreak preparedness and response in Africa

This unified continental approach marks a pivotal step towards achieving greater alignment, harmonisation and integration in Africa’s health response efforts – essential on a continent with limited resources to effectively prevent, detect and respond to health threats. By consolidating the efforts of key organisations, this model reduces the reporting and planning burdens that often consume the time of frontline health workers, freeing them to focus on critical preparedness and response activities.

This unified approach also enhances the efficiency of the CMPRP, streamlining processes for better resource allocation and execution. For example, community health workers involved in mpox preparedness will undergo a single, modular training that builds their capacity on risk communication and community engagement, surveillance, IPC, vaccination and home care, which not only saves time and money but also reinforces consistency in care delivery. In addition, having key partners work together allows for unified communication, cohesive support and improved synergy.

This response represents the first instance of using a single continental coordination team, response plan, budget and M&E framework in a major health emergency. Its success hinges on partners’ commitment to a collective rather than organisation attribution, keeping the welfare and well-being of the people at the forefront. Ultimately, this model aims to build a more resilient health system across the continent, one capable of responding swiftly and effectively to future health crises. This approach, if sustained, can transform Africa’s public health landscape by reinforcing preparedness, equity and local leadership in global health responses.
